# Implication of Soluble Forms of Cell Adhesion Molecules in Infectious Disease and Tumor: Insights from Transgenic Animal Models

**DOI:** 10.3390/ijms19010239

**Published:** 2018-01-13

**Authors:** Etsuro Ono, Toshimitsu Uede

**Affiliations:** 1 Department of Biomedicine, Center of Biomedical Research, Graduate School of Medical Sciences, Kyushu University, Fukuoka 812-8582, Japan; 2 Division of Molecular Immunology, Institute for Genetic Medicine, Hokkaido University, Sapporo 060-0815, Japan; uedetoshimitsu@gmail.com

**Keywords:** cell adhesion molecule, nectin-1, nectin-2, herpesvirus entry mediator (HVEM), Siglec-9, disease-resistant, therapeutic agent

## Abstract

Cell adhesion molecules (CAMs) are surface ligands, usually glycoproteins, which mediate cell-to-cell adhesion. They play a critical role in maintaining tissue integrity and mediating migration of cells, and some of them also act as viral receptors. It has been known that soluble forms of the viral receptors bind to the surface glycoproteins of the viruses and neutralize them, resulting in inhibition of the viral entry into cells. Nectin-1 is one of important CAMs belonging to immunoglobulin superfamily and herpesvirus entry mediator (HVEM) is a member of the tumor necrosis factor (TNF) receptor family. Both CAMs also act as alphaherpesvirus receptor. Transgenic mice expressing the soluble form of nectin-1 or HVEM showed almost complete resistance against the alphaherpesviruses. As another CAM, sialic acid-binding immunoglobulin-like lectins (Siglecs) that recognize sialic acids are also known as an immunoglobulin superfamily member. Siglecs play an important role in the regulation of immune cell functions in infectious diseases, inflammation, neurodegeneration, autoimmune diseases and cancer. Siglec-9 is one of Siglecs and capsular polysaccharide (CPS) of group B *Streptococcus* (GBS) binds to Siglec-9 on neutrophils, leading to suppress host immune response and provide a survival advantage to the pathogen. In addition, Siglec-9 also binds to tumor-produced mucins such as MUC1 to lead negative immunomodulation. Transgenic mice expressing the soluble form of Siglec-9 showed significant resistance against GBS infection and remarkable suppression of MUC1 expressing tumor proliferation. This review describes recent developments in the understanding of the potency of soluble forms of CAMs in the transgenic mice and discusses potential therapeutic interventions that may alter the outcomes of certain diseases.

## 1. Introduction

A number of cell adhesion molecules (CAMs), mediate homophilic (single type) and heterophilic (different type) cell adhesions. The CAMs control the cell behavior including differentiation, proliferation and migration by modulation of expression levels of the CAMs on the cells. In a heterophilic interaction, there are also carbohydrate chain-binding lectin molecules in mucin-like CAMs on adjacent cells.

Modulation of surface expression by endocytosis of the CAMs is mediated by disruption of the CAM homodimer interface. Virus entry into the cells using this phenomenon is thought to be an example of the CAM binding phenotype. Many diverse groups of viruses bind to immunoglobulin super family CAMs at the cell surface to mediate cell entry. It is expected that a detailed understanding of processes of virus attachment and entry will provide researchers with new targets for development of anti-viral agents. In particular, mechanisms found to be widespread among diverse groups of viruses, such as entry via immunoglobulin super family CAMs binding, are appealing targets as interventions have the potential for broad-spectrum activity [1].

Nectins are important cell–cell adhesion molecules and belonging to Ca^2+^-independent immunoglobulin superfamily [2]. The nectin family has four members, nectin-1, -2, -3 and -4 and some of them have two or three splicing variants [3]. In general, nectin molecule is made of an extracellular region containing three Ig-like loops, a single transmembrane region and a cytoplasmic tail. The extracellular regions consist of a single V-like domain (first Ig-like domain) and two C-like domains and form homo-*cis*-dimers for promoting homophilic or heterophilic *trans*-interactions. As a key molecule, nectins are concerned with the organization of adherens and tight junctions in epithelial and endothelial cells. Nectin-1, nectin-2 and nectin-3 localize in cadherin-based adherens junctions in epithelial cells and their cytoplasmic tails bind to PDZ domains of afadin, the actin filament-binding protein and components of the junctions [4,5,6,7,8]. In a broad range of tissues and cells, nectin-1 and nectin-2 are expressed [9,10,11]. Human nectin-1 gene is mutated in cleft lip and palate/ectodermal dysplasia 1 syndrome (CLPED1), and its mutations have been associated with non-syndromic cleft lip with or without cleft palate (NSCL/P) [12]. This protein mediates viral entry into cells of herpes simplex viruses 1 and 2 (HSV-1, HSV-2), and pseudorabies virus (PRV) as a receptor for their glycoprotein D (gD) [13,14].

Herpesvirus entry mediator (HVEM), also known as tumor necrosis factor receptor superfamily member 14 (TNFRSF14), is a cell surface receptor of the TNF-receptor superfamily. This receptor was identified as a cellular mediator of HSV-1 and HSV-2 entry. Binding of the viral envelope glycoprotein D (gD) of HSVs to this receptor protein has been shown to be part of the viral entry mechanism. HVEM is expressed in lymphoid cells but also in other cell types [15,16]. Mice encode a related protein that is only 45% identical to the human form [17]. Both human and mouse forms of HVEM can mediate entry of HSV-1 and HSV-2 but have no entry activity for PRV and bovine herpesvirus type 1 (BHV-1) [16,18]. Natural ligands for HVEM are members of the TNF family including lymphotoxin-α (LTα) and a cellular ligand for herpesvirus entry mediator and lymphotoxin receptor (LIGHT) [19]. Through an interaction with HVEM, LIGHT presumably stimulate the proliferation of T cells [15,20]. The cytoplasmic tail of HVEM transmits signals through TNF receptor-associated factors (TRAFs) for activation of AP1 and NF-κB1, for example [17,21]. Deletion of the cytoplasmic tail of HVEM did not have an influence on the receptor ability of HVEM for HSV [16]. Although signal transduction after HSV entry may influence in viral replication or virus–cell interaction, it seems not necessary for virus entry.

Siglecs are sialic acid-binding immunoglobulin-like receptors, and play a role in discrimination of “self” and “non-self” [22]. In addition, binding of Siglecs to their ligands containing sialic acids regulates the innate and adaptive immune systems [22]. However, Siglecs also function as a critical host defense receptor to restrict invasive bacterial infection since they recognize the sialic acid epitope utilized by the sialylated pathogens to suppress host innate immune responses. Siglec-9 is an immune inhibitory receptor and binds to its ligands to induce inhibition of anti-tumor immunity of NK cell and T cell [23,24]. It is also interestingly that binding of tumor-produced MUC1 to Siglec-9 induces negative immunomodulation [25].

Soluble forms of nectin-1 and HVEM bind to gD of alphaherpesviruses. On the other hand, the soluble form of Siglec-9 binds to sialic acid of bacteria and tumors. By using these properties, their anti-viral, bacterial and tumor potentials in transgenic mice have been shown. A brief overview of transgenic animal models expressing soluble forms of the CAMs used to evaluate their potential in the viral and bacterial infections and a tumor as well as a discussion on the possible therapeutic usage and application for disease-resistant animals will be also provided.

## 2. Soluble Forms of Nectins

The nectin family is related to poliovirus receptor in sequence and structure. Primary sequence of porcine, human and mouse nectin-1 are highly conserved. Porcine and human nectin-1 have 96% amino acid identity and nectin-1 from African green monkey, mouse, hamster, and cow are similarly conserved [26]. Porcine nectin-1 mediates entry of HSV-1, HSV-2, PRV and BHV-1 [26]. It seems likely that various mammalian forms of nectin-1 will prove to be pan-alphaherpesvirus entry receptors. Nectin-1 and nectin-2 appear to be expressed by a broad range of tissues and cells [8,9,10]. The earlier studies reported that soluble forms of human and porcine nectin-1 blocked infection of HSV-1 in addition to PRV and BHV-1 [10,27].

### 2.1. Porcine Nectin-1

The transgenic mouse lines expressing a soluble form of porcine nectin-1 (pNectin-1-hIg; VCC-hIg), consisting of the entire ectodomain of porcine nectin-1 and the Fc portion of human IgG1 showed nearly complete resistance to PRV infection by means of both intraperitoneal and intranasal routes [28]. It was especially noteworthy that protection against PRV entry in the sites of primary infection was observed in the transgenic mice after intranasal inoculation of PRV. The transgenic embryonic fibroblasts expressing VCC-hIg showed marked resistance to PRV infection. PRV DNA in the trigeminal ganglia was not detected in the transgenic mice inoculated with the attenuated PRV by means of the intranasal route. Furthermore, no specific antibody to PRV was found in the surviving transgenic mice. VCC-hIg was expressed in the transgenic nasal mucosa and respiratory tracts. Taken together, expression of VCC-hIg protected transgenic mice against PRV primary infection and not only against disease symptoms, and the epithelial cells of the nasal mucosa and respiratory tracts expressing VCC-hIg were also resistant to PRV infection.

### 2.2. Comparison of the Antiviral Potentials among Different Types of Soluble Forms of Porcine Nectin-1

**Figure 1:**
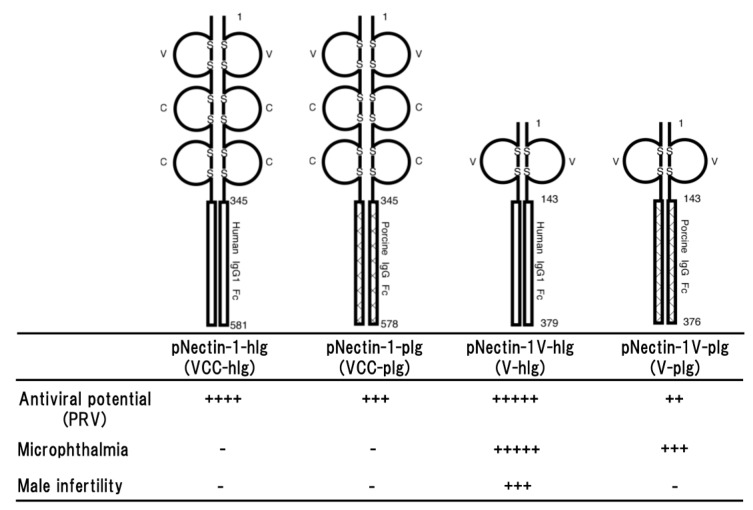
Schematic representation of four different types of soluble forms of porcine nectin-1. The fusion proteins consist of the entire ectodomain (VCC) or the first Ig-like (V) domain of porcine nectin-1 and Fc portion of human IgG1 or porcine IgG (upper part) and the properties of four transgenic mouse lines (lower part). Antiviral potential against PRV infection [29,31], microphthalmia and male infertility [34] as a side effect are shown. Numbers on the figure are shown amino acids number of each molecule. S represents disulfide bond. + to +++++ indicate relative strength of the phenotype. − means no phenotype observed.

The primary effect of the fusion protein is thought to bind to the virus and promote its clearance by macrophage via the Fc portion. However, it is also considered that functional differences between human and porcine Fc portions may be appeared in a murine immune system. In addition, there may be also the role of the second and third domains of porcine nectin-1 in the antiviral potential against PRV infection. The gD-binding domain of nectin-1 is the first or N-terminal immunoglobulin (Ig)-like domain of the entire ectodomain. Therefore, four different types of transgenic mouse lines were compared in resistance against PRV infection. The fusion proteins were consisting of the entire ectodomain or the first Ig-like domain of porcine nectin-1 and the Fc portion of porcine or human IgG; pNectin-1-hIg (VCC-hIg), pNectin-1-pIg (VCC-pIg), pNectin-1V-hIg (V-hIg) and pNectin-1V-pIg (V-pIg) (Figure 1).

Direct comparison of the effects of Fc portions from human and porcine IgG demonstrated that there was not much difference between them in antiviral potential [29]. However, it seemed that the antiviral potential of VCC-hIg made of the entire ectodomain fused to the human Fc portion was slightly stronger than that of VCC-pIg made of the entire ectodomain fused to the porcine Fc portion. Transgenic mice expressing VCC-pIg were more resistant than those expressing V-pIg made of the first Ig-like domain fused to the porcine Fc portion [29], although the first immunoglobulin-like domain of porcine nectin-1 was sufficient to confer resistance to PRV infection in transgenic mice [30]. Surprisingly, the animals expressing V-hIg made of the first Ig-like domain of porcine nectin-1 fused to the human Fc portion showed marked resistance to PRV challenge via both intraperitoneal and intranasal routes. It was especially noteworthy that protection against PRV entry in the sites of primary infection was almost complete in the transgenic mice after intranasal inoculation of PRV [31]. In addition, the resistance of the transgenic mice was the best among four different types of transgenic mouse lines. The high resistance was surprising, as V-pIg and, despite comparable expression levels, did not provide such a high resistance to the transgenic mice [29]. Altogether, the fusion protein consisting of the first Ig-like domain fused to the human Fc portion provided marked resistance against PRV infection to the transgenic mice (Figure 1).

In comparison between VCC-hIg and V-hIg or VCC-pIg and V-pIg, it was found that two C domains affected their antiviral potentials, indicating that two C domains provide a certain degree of flexibility to the first Ig-like (V) domain for suitable interaction with gD and/or enhance the binding affinity to gD. It was reported that the second and third C domain of nectin-1 increased the efficiency of entry of HSV-1 gD [32]. On the other hand, in comparison between VCC-hIg and VCC-pIg or V-hIg and V-pIg, a remarkable difference was observed in that of V-hIg and V-pIg, but not VCC-hIg and VCC-pIg. Although there may be functional differences between whole human and porcine Fc portions in the murine immune system, human Fc portion did not confer a significant difference in the mouse immune response, at least when the transgenic mice expressing a fusion protein made of an entire ectodomain of HVEM fused to the human Fc portion were immunized with ovalbumin [33]. The remarkable difference in V-hIg and V-pIg may be due to conformational difference of two molecules, but not functional difference of Fc portions. Further investigation of the interactions among each soluble form of nectin-1, endogenous nectins and gD should be necessary for elucidation of the antiviral mechanisms of soluble forms of nectin-1.

### 2.3. Side Effects Observed in Transgenic Mice

Nectin-1 is an important molecule of intercellular junctions and plays a critical role in morphogenesis, differentiation, proliferation, and migration. Nectin-1 is also concerned with junctional disorders associated diseases. Therefore, careful analyses of transgenic mice are essential for the issue of possible adverse effects. In the transgenic mouse lines expressing VCC-hIg or VCC-pIg, any differences in detailed histological examination of main tissues was not induced by expression of the fusion proteins [28,29]. Functional neuronal disorder such as motility disturbance was not observed at least in the rotarod test. They developed with normal body weights and state of health was normal, and litter sizes were the same as those of wild type. Taken together, overexpressing the soluble form of porcine nectin-1 in mice may not raise host safety issues (Figure 1).

On the other hand, all V-hIg mice showed microphthalmia and small vitreous cavity, and V-hIg was co-localized with the endogenous nectins in the developing optic nerve of the V-hIg mice [34]. It is interesting that studies on mice lacking nectin-1 or nectin-3 showed a virtually identical ocular phenotype: microphthalmia [35]. In addition, careful examination of V-pIg mice revealed that the vitreous body was also absent or few in approximately 40% of all examined eyes [34]. The defect observed in V-pIg mice was not as severe compared with that in V-hIg mice. In the eyes of the VCC-hIg transgenic mice, abnormality was not observed, although VCC-hIg mice showed marked resistance to PRV infection, as V-hIg mice and the virus neutralization titers of sera from VCC-hIg mice were much higher than those of sera from V-hIg mice. This discrepancy in the abnormality of transgenic eyes between the V-hIg and VCC-hIg mice may be due to the difference in the affinity between the two fusion proteins to the molecule that is involved in microphthalmia, but not the virus neutralization. In the detailed histological examination of the eyes, it was revealed that V-hIg strongly bound to intact nectins, which are involved in the development of the eye, and caused microphthalmia [34]. Studies on mice lacking nectin-1 or nectin-3 showed a virtually identical ocular phenotype: microphthalmia [34]. In addition, it is also interesting that male infertility was observed in approximately 60% of the transgenic founder mice expressing V-hIg as in nectin-2^−/−^ and nectin-3^−/−^ mice [35,36]. Furthermore, there is a report to support a possibility that V-hIg binds to intact nectins. Fabre et al. reported that the first Ig-like V domain of nectin-1 inhibited a homophilic or heterophilic trans-interactions of intact nectins on the cells [37]. Taken together, as a dominant-negative mutant, V-hIg interacted with the intact nectins on the cell, leading to impairment of ocular development, spermatogenesis, although the exact mechanism remains to be elucidated (Figure 1).

### 2.4. Possible Mechanism of Resistance against PRV Infection

There are several possible effects of soluble forms of porcine nectin-1 on the suppression of virus replication. Firstly, cell-bound fusion proteins may inhibit the virus entry into cells. Secondly, intracellular fusion proteins may bind to newly synthesized glycoprotein D and inhibit secondary infections as intrabodies [38,39]. Thirdly, secreted fusion proteins may inhibit secondary infections of the cells, neutralizing free virus released in the first round of infection. Fourthly, cell-bound and intracellular fusion proteins may inhibit secondary infection in the cells mediated by cell-to-cell spread. These possible effects of the fusion proteins, expressed in the nasal mucosa and respiratory tract, may account for the protection against intranasal PRV infection. 

There are several reports supporting mechanism to have the possible effects mentioned above except the third effect. Firstly, V-hIg and endogenous nectin-1 were co-localized at cell–cell junctions of nasal squamous epithelium [34]. Secondly, survival rate of the V-hIg mice was comparable or higher than that of the VCC-hIg mice, although neutralization titers of sera from V-hIg mice were much lower than those of sera from VCC-hIg mice, supporting the mechanisms other than direct binding of the fusion proteins to gD. Thirdly, cell-bound VCC-hIg inhibited virus entry into cells, because the cells expressing VCC-hIg still showed significant resistance, even when the secreted VCC-hIg were washed away [28,40]. Fourthly, a fusion protein made of the first Ig-like domain of nectin-1 fused to the human Fc portion inhibited a homophilic or a heterophilic trans-interaction of extracellular regions of all nectins on the cells [37]. Altogether, soluble forms of porcine nectin-1 interacted with intact nectin-1 on the cell and interrupted the binding of gD to nectin-1, leading to inhibition of virus entry into the cell. Fifthly, it is also interestingly that newly synthesized gD binds to nectin-1 of the non-infected cell and maintains nectin-1 at junctions in the site suitable for virus spread [41]. This supported the mechanism that soluble forms of nectin-1 interacting with endogenous nectin-1 may sequester the newly synthesized gD, resulting in the inhibition of viral spread.

### 2.5. Application for Development of Pseudorabies-Resistant Pig

PRV is one of pathogens causing major economic risks in the swine industry worldwide. In PRV infection, abortion and infertility were often observed in breeding sows and also acute respiratory syndrome in growing pigs. In piglets, PRV causes lethal encephalitis leading to death. In all surviving pig, latent infection was established and they became reservoirs. In general, vaccination strategy alone is not able to eradicate the disease and usually only suppress manifestation of the disease.

Agents targeted at gD mediating the viral entry are of great potential to be anti-alphaherpesvirus agents. As described above, expression of soluble forms of cellular receptors for gD provided a significant resistance against alphaherpesvirus infection to cell lines and transgenic mice [28,29,30,31,33,40]. Comparison of the antiviral potentials among the pseudorabies-resistant transgenes revealed that the transgene encoding a soluble form of the entire ectodomain of porcine nectin-1 fused to the Fc portion of human IgG1 (pNectin-1-hIg; VCC-hIg) was the best without any side effects in mice in appearance [28,29,31]. As an alternative strategy, this in vivo model system may be applied for generation of farm animals with enhanced resistance to pseudorabies.

Two of five transgenic pig lines expressing VCC-hIg showed resistance against PRV infection. In the transgenic pigs, body temperature, body weight and manifestation of the disease were significantly suppressed. The piglets from both lines showed significant resistance to PRV lethal infection via intranasal routes; 90% (10/11) and 50% (6/12) piglets from each line were survived, respectively. This was the first example of transgene-mediated genetic immunization in farm animals.

### 2.6. Human Nectin-1

**Figure 2:**
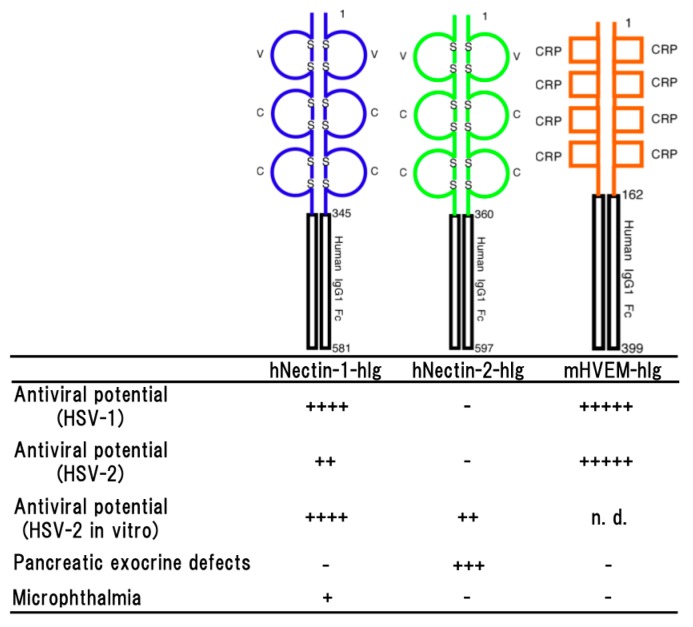
Schematic representation of a soluble form of human nectin-1 (hNectin-1-hIg), human nectin-2 (hNectin-2-hIg) and murine HVEM (mHVEM-hIg). Each fusion protein consists of each entire ectodomain and Fc portion of human IgG1 (upper part) and the properties of each transgenic mouse line (lower part). Antiviral potential against HSV-1 [33,44], HSV-2 in vivo [44] or in vitro [48] infection, pancreatic exocrine defects [48] and microphthalmia (unpublished data) as a side effect are shown. Numbers on the figure are shown amino acids number of each molecule. S represents disulfide bond. + to +++++ indicate relative strength of the phenotype. − means no phenotype observed.

Human nectin-1 is competent receptor for both HSV-1 and HSV-2 entry, while nectin-2 is a receptor for HSV-2 and not for wild-type of HSV-1 [11,42,43]. The transgenic mouse lines which effectively expressing hNectin-1-hIg made of the entire ectodomain of human nectin-1 and the Fc portion of human IgG1 showed significant resistance against both of HSV-1 and HSV-2 infections (Figure 2) and the difference in survival rates among them was dependent on the expression level of hNectin-1-hIg [44]. It was reported that vaginal infection with HSV-2 was blocked by pre-incubation of the virus with soluble recombinant nectin-1 [45]. A neutralization assay using the sera of transgenic mice line also showed that over 90% of plaque formation of HSV-2 was inhibited, demonstrating that hNectin-1-hIg has an effective antiviral potential. However, in the experimental infections, only 50% of the transgenic mice survived against HSV-2 infection, although the concentration of hNectin-1-hIg in sera was enough for the viral neutralization. It has been reported that HSV-2 strains tend to cause more severe disease in animal models than HSV-1 strains [46,47]. Therefore, this may be considered the reason for apparent resistance in the transgenic mice to HSV-2 infection not being observed. On the other hand, almost all of the transgenic mice survived until the end of the experimental period of infection with HSV-1. These findings suggested that the antiviral effect of hNectin-1-hIg is more efficient against HSV-1 infection than HSV-2 infection in vivo.

As a side effect, same microphthalmia as shown in pNectin-1V-hIg (V-hIg) mice was observed in the higher expresser of the transgenic mouse lines expressing hNectin-1-hIg (Figure 2), but not in lower expresser (unpublished data). Since the microphthalmia was not seen in any transgenic mouse lines expressing pNectin-1-hIg (VCC-hIg), this discrepancy in the abnormality of transgenic eyes between the hNectin-1-hIg and pNectin-1-hIg mice may be due to the difference in the affinity between the two fusion proteins to the molecule, which is involved in microphthalmia. In mice, hNectin-1-hIg may have a higher affinity to the molecule as compared with that of pNectin-1-hIg.

### 2.7. Human Nectin-2

Human nectin-2 can serve as an entry receptor for HSV-2, but not murine nectin-2 [43]. Although it has been demonstrated that expressing nectin-2 in various human cell types of neuronal, fibroblastic and epithelial origin is able to mediate the entry of HSV-2 into cultured cell lines [11,43]. The transgenic mice expressing hNectin-2-hIg made of the entire ectodomain of human nectin-2 and the Fc portion of human IgG1 did not show any resistance against the HSV-2 infection [44], although the expression level of hNectin-2-hIg was significant and effective enough, demonstrating that hNectin-2-hIg did not show the antiviral effect against HSV-2 infections in vivo (Figure 2).

The transgenic mice expressing hNectin-2-hIg did not show resistance against HSV-2 infection, whereas, in vitro, it was reported that hNectin-2-hIg could exert the suppression effect of HSV-2 replication in transformed Vero cells [49]. There may be a possible explanation for this discrepancy. In in vitro experiments, concentration-dependent viral resistance of hNectin-2-hIg accumulating into the transformed cell lines was observed, suggesting that intracellular accumulation of hNectin-2-hIg over a definite amount was responsible for exertion of the antiviral effect [49]. However, the proliferation status of mucosal cells in the abdominal cavity of the transgenic mice was not synchronized because of the different life spans of various cell types. Therefore, it seemed likely that accumulation of nectin-2Ig in all types of mucosal cells of the transgenic mice was not enough to prevent the viral replication, and consequently the mice were killed by the HSV-2 infection.

In the transgenic mice expressing hNectin-2-hIg, side effects were observed such as distinctive elevation of serum amylase and lipase [48]. Histopathologically, it was suggested that hNectin-2-hIg was continuously expressed in the cytoplasm including the zymogen granules, resulting in impairment of zymogen granule formation. It seemed that hNectin-2-hIg impaired exocrine secretion of pancreas and formation of zymogen granules in transgenic mice (Figure 2). The most likely intracytoplasmic accumulation of abnormal proteins disrupted cytoarchitectures and functions of the acinar cells, following the elevating of amylase and lipase levels in the sera.

## 3. Herpesvirus Entry Mediator (HVEM)

HVEM is expressed in mainly lymphoid cells, but the expression is observed in other cell types [15,16]. Although mouse HVEM is only 45% identical to the human form [17], as with human HVEM, mouse HVEM can also mediate entry of HSV-1 and HSV-2, but not PRV and BHV-1 [16,18].

### 3.1. HSV-1

The transgenic mice which effectively expressing mHVEM-hIg made of the entire ectodomain of mouse HVEM and the Fc portion of human IgG1 showed a significant resistance against HSV-1 infection [33] (Figure 2). As reported previously [10,50,51], transgenic mouse sera containing mHVEM-hIg blocked HSV-1 infection in vitro and their embryonic fibroblasts showed marked resistance against HSV-1 infection. These findings suggested that mHVEM-hIg played an important role on the resistance against HSV-1 infection in transgenic mice. In intravenous infection with HSV-1, almost complete protection was observed, suggesting a possibility that the primary effect of the fusion protein was to bind to virus in serum and promoted its clearance by macrophage via the Fc portion of human IgG1. In the survived transgenic mice except for only one mouse, latency-associated transcripts (LATs) was not detected in the trigeminal ganglia, indicating that latent infection was not established and protection was almost complete. Likely, only one mouse expressing LATs was infected with HSV-1, suggesting that mHVEM-hIg may suppress manifestation of the disease and let the mouse survive, as a result. In addition, it seemed likely that the resistance was dependent on the expression level of mHVEM-hIg.

HVEM is a member of TNF receptor family and a receptor for LTα and LIGHT [19] and plays an important role on the immune systems. Because mHVEM-hIg can also block the binding between HVEM and LIGHT, mHVEM-hIg may affect the host immune responses against the insulting agents. Although the possibility could not be eliminated completely, significant difference in the immune responses was not observed, at least in comparison between the transgenic and non-transgenic mice immunized with ovalbumin. It seemed likely that fatal disturbances of the immune responses did not occur in the transgenic mice [33]. In addition, the transgenic mice developed normally and there was no difference in the body weights of the transgenic and non-transgenic mice, and no gross abnormality was observed (Figure 2).

### 3.2. HSV-2

It was reported that activities of HSV-2 cell entry mediated by murine HVEM and nectin-1 were indistinguishable from those of human HVEM and nectin-1 [52]. However, human nectin-2 could serve as an entry receptor of HSV-2, but not murine nectin-2 [43]. Furthermore, murine HVEM, but not human HVEM, can bind to murine LIGHT, which is a natural ligand for HVEM and regulates T cell immune responses [53]. It was also reported that HSV-2 infection in the vaginal epithelium required expression of either nectin-1 or HVEM, because HSV-2 could not infect nectin-1/HVEM double knockout mice [54]. Interestingly, the absence of nectin-1, but not HVEM, reduced efficiency of HSV-2 spread to the nerve system via both vaginal and intracranial routes of infection [54,55]. However, the resistance to HSV-2 infection in the mice expressing mHVEM-hIg was significantly higher than in the mice expressing hNectin-1-hIg and hNectin-2-hIg, although expression levels of both hNectin-1-hIg and hNectin-2-hIg were higher than that of mHVEM-hIg [44], indicating that mHVEM-hIg was more effective than hNectin-1-hIg and hNectin-2-hIg for protection against HSV-2 infection in transgenic mice (Figure 2).

The transgenic mice expressing mHVEM-hIg showed even more remarkable resistance against HSV-2 infections, but their uninfected sera did not inhibit the viral infection in vitro [44], indicating that neutralization with mHVEM-hIg was not concerned with the antiviral mechanism observed in the mice expressing mHVEM-hIg. Krummenacher et al. [56] reported that all clinical isolates of HSV-2 have the ability to use HVEM as a receptor despite the fact that it is expressed mainly on the immune systems, which are not targets of productive HSV or latency in vivo. The principal natural function of HVEM is that it is known to regulate the T cell development signal (LIGHT signaling) through interaction with LIGHT, which is expressed in activated lymphocytes, natural killer cells and immature dendritic cells [57]. LIGHT signaling appears to be a strong potential candidate molecule to facilitate negative selection of T cells [57]. Wang et al. [58] reported that suppression of LIGHT signaling by mHVEM-hIg inhibited negative selection, resulting in an increase in the percentage of CD^4+^ CD^8+^ double-positive and CD^8+^ single-positive cells. In the transgenic mice, mHVEM-hIg might compete the interaction of innate HVEM with LIGHT, with the result that strongly activated immune cells proliferated enough to overcome the HSV-2 infections. In fact, serological examination demonstrated that establishment of HSV-2 infection was confirmed in all of the surviving transgenic mice infected with HSV-2, but no symptoms were observed [44]. Suppression of manifestation of the disease was also observed in the transgenic mice expressing mHVEM-hIg infected with HSV-1 [33]. These observations may support the hypothesis that an activated immune response by mHVEM-hIg is responsible for the resistance to HSV-2 infection in the transgenic mice expressing mHVEM-hIg. To elucidate the mechanism of antiviral effects of mHVEM-hIg, further investigation is needed.

Genital HSV-2 infection causes significant morbidity [59] and is an important cofactor for HIV-1 infection [60,61,62]. Furthermore, in the past two decades, investigations have amply documented the increase in the frequency of genital HSV-1 compared with genital HSV-2 infection [63,64,65]. A vaginal microbiocide able to protect against HSVs transmission could contribute significantly to controlling sexually transmitted diseases. New additional prevention and therapeutic strategies against both HSV-2 and HSV-1 infections are highly desirable. Since the remarkable antiviral potential of mHVEM-hIg was observed in transgenic mice, a soluble form of human HVEM may be a candidate as a new medical agent against HSV infection and gene-based therapy in chronic HSV patients, although drug efficacy studies in mouse models are not always translated to human trials.

## 4. Siglec-9

Siglecs are sialic acid-binding immunoglobulin-like receptors, and play a role in discrimination of “self” and “non-self” and regulate the innate and adaptive immune systems by binding to the ligands containing sialic acids [22].

However, Siglecs also function as a critical host defense receptor to restrict invasive bacterial infection since it recognizes the terminal sialic acid epitope utilized by the sialylated pathogens to suppress host innate immune responses. So far, one of the best evidence that bacterial sialic acids can subvert immune responses via Siglec interactions has come from studies of Group B *Streptococcus* (GBS, *S. agalactiae*). Engagement of a α 2–3-linked sialylated capsular polysaccharide (CPS) of GBS serotype III through Siglec-9 of neutrophils subverts the host innate immune response by transmitting negative regulatory signals and dampens neutrophil responses. In addition, the engagement also promoted evasion of neutrophil-based GBS killing and increased bacterial survival [66].

In addition, Siglec-9 as an immune inhibitory receptor binds to its ligands and inhibits anti-tumor immunity of NK cell and T cell [23,24]. Interestingly, binding of tumor-produced MUC1 to Siglec-9 induces negative immunomodulation [25].

### 4.1. Group B Streptococcus

GBS is a Gram-positive encapsulated bacterium and existing in the vagina of 15–30% of healthy women. GBS causes neonatal pneumonia, meningitis and septicemia [67,68,69]. The GBS colonization raises premature delivery and preterm rupture of membranes during pregnancy [70,71]. GBS expresses CPS that is a major virulence factor contributing to evasion of host immune defense mechanisms [72], promoting GBS survival in vivo [73,74] and interfering with host complement system functions [75,76]. GBS CPS can be divided into ten serotypes (Ia, Ib, II–IX) by the unique antigenic and structural features [77]. Nevertheless, all the GBS CPS possessing repeating units containing a terminally capped sialic acid as a critical conserved element.

This bacterium displays Siaα2–3Galβ1–4GlcNAc moieties on its capsule that are recognized by Siglec-9 on human neutrophils and its murine equivalent, Siglec-E [66,78]. Carlin et al. [66] provided the first in vitro evidence that GBS dampens neutrophil activation by engaging the inhibitory human Siglec-9, resulting in impaired oxidative burst and neutrophil extracellular trap (NET) formation, thereby reducing neutrophil bactericidal activity. Following intranasal infection with GBS, Siglec*E* knock out mice produced higher levels of the proinflammatory cytokines interleukin-1β (IL-1β) and IL-6, but reduced levels of IL-10 [78]. Although no significant overall difference in the blood GBS number was observed between wild type and Siglec*E* knock out mice when a sub-lethal dose of GBS was systemically challenged, Siglec*E* knock out mice showed reduced bacterial dissemination in the brain and kidney, which is consistent with bacterial exploitation of Siglec-dependent inhibitory pathways. Thus, Siglec-9 was found to be a key in control of invasive GBS infection even within the in vivo milieu.

**Figure 3:**
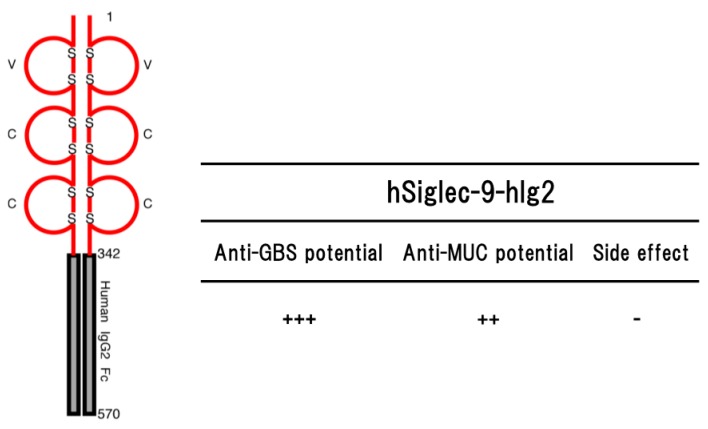
Schematic representation of a soluble form of human Siglec-9 (hSiglec-9-hIg2) consisting of the ectodomain of human Siglec-9 and Fc portion of human IgG2 (left part) and their potential (anti-GBS [79] and anti-MUC [88]) in the transgenic mice are shown (right part). Numbers on the figure are shown amino acids number of each molecule. S represents disulfide bond. ++ to +++ indicate relative strength of the phenotype. − means no phenotype observed.

The transgenic mice expressing hSiglec-9-hIg2 showed significant resistance to lethal infection with GBS serotype III [79] (Figure 3). The resistance was due to the function of hSiglec-9-hIg2 expressed in the transgenic mice, but not the function of immunological memory induced by vaccination or natural infection before challenge. The hSiglec-9-hIg2 was consisting of the ectodomain of human Siglec-9 (amino acids 1–342) and Fc portion of human IgG2. Part of the ectodomain of human Siglec-9 constituting hSiglec-9-hIg2 bound to the sialylated-GBS strains of serotypes Ia, Ib, II, III, IV and V in whole GBS cell ELISA, indicating that the binding of sSiglec-9 to CPS of GBS serotype III interfered with the transmission of immunosuppressive signal and rescued host complement system functions, and/or thereby removing the survival advantage to the GBS. As the functions of the Fc portion constituting hSiglec-9-hIg2, it is thought that there are two important effecter functions of Fc portion of IgG called opsonization and/or complement activation [80]. Human IgG2 activates both murine NK cells and murine polymorphonuclear neutrophils (PMNs), but not murine macrophages [81], suggesting that hSiglec-9-hIg2 could induce the Fc-mediated phagocytosis. It is also possible that the binding of hSiglec-9-hIg2 to CPS on the surface of GBS triggers subsequent activation of the complement cascade, leading to the formation of the membrane attack complex (MAC) at the surface of GBS and facilitating MAC-mediated lysis, although IgG2 antibodies are effective mainly at high epitope density [82,83]. Thus, hSiglec-9-hIg2 may be able to enhance the bacterial clearance through the innate immune mechanism utilizing the Fc portion of human IgG2.

Late-onset GBS disease (LOD) occurs within the first 90 days of life. It is characterized by meningitis, which was observed in up to 50% of cases [68]. Unlike early-onset GBS disease (EOD) pathogenesis, LOD may be provided from mother’s milk or the nosocomial and the community source in the perinatal period. Prematurity is thought to be the main risk factor for developing LOD and the risk is proportionally increased for every week of prematurity [84]. LOD is caused by GBS serotype III mainly. The mortality rate of LOD is lower than EOD but morbid state is high, because approximately 50% of newborn babies who survived LOD GBS infectious disease suffered from neurological deficit such as hearing loss, speech and language delay, and mental retardation [68,85]. EOD was effectively decreased by use of intrapartum antibiotic prophylaxis (IAP) [86]. In contrast, IAP was not much effective to the incidence of LOD. Although significant advances in global public heath care of neonatal disease were achieved, sepsis and meningitis caused by GBS are still present [87]. Therefore, further prevention and therapeutic strategies to the infectious disease are highly desirable [87]. In the transgenic mouse lines, histopathological, immunological, at least a number of lymphocyte homing into lymph nodes, and blood biochemical abnormalities were not observed [88]. Since hSiglec-9-hIg bound to CPS of GBS serotype III, it might lead to inhibit the bacterial infection at the primary site of infection.

Taken together, hSiglec-9-hIg2 provides an antibacterial benefit against GBS serotype III infection in the transgenic mice mediated by interfering with the down-regulation of the immune responsiveness of neutrophils, rescuing host complement system functionality, thereby removing the survival advantage to the GBS, and also possibly by enhanced bacterial clearance through the innate immune mechanism utilizing the Fc portion of human IgG2 constituting hSiglec-9-hIg2. Thus, hSiglec-9-hIg2 could restrain propagation of GBS serotype III, and might be a candidate as a new medical agent against LOD.

### 4.2. MUC1 Tumor

The MUC1 is one of major sialylated transmembrane glycoproteins and cleaved into two subunits (MUC1-N and MUC1-C) and the two subunits form a heterodimer on the cell membrane [89,90,91]. The MUC1 is expressed at the apical surface of epithelial cells to protect from the external environment [92,93] and overexpressed in various adenocarcinoma including breast carcinomas [92,94,95]. As a functional role, the MUC1 precipitates in the tumor growth, tumorigenicity, and resistance to apoptosis, and it relates to poor-prognosis of the tumors [96,97,98,99,100,101]. It has shown that MUC1-C interactions with EGFR, ErbB2, and other receptor tyrosine kinases and functions as an oncoprotein [102]. In addition, MUC1 also directly bind to β-catenin, and blocks phosphorylation and degradation, contributing to the malignant phenotype of tumors [103]. Therefore, MUC1 is thought to be a therapeutic target for development of anti-tumor agents. Many agents that directly target MUC1 including anti-MUC1 monoclonal antibody and anti-MUC1 vaccines have been developed [102,104,105,106,107,108,109,110]. However, a role of MUC1 in malignant tumor growth mechanism is not elucidated yet.

It was reported that tumor-produced mucins bound to Siglec-9, leading to negative immunomodulation [25]. It is known that Siglec-9 is an inhibitory receptor expressed on immune cells and the binding of Siglec-9 to its ligands induces inhibition of NK cell and T cell antitumor immunity [23,24]. Therefore, it was expected that an agent inhibiting the binding of Siglec-9 to MUC1 interfered the transmission of immunosuppressive signal, leading to provide anti-tumor potential to the animals bearing MUC1-expressing tumors. However, there was a report that binding of the soluble form of Siglec-9 to the MUC1 induced recruitment of β-catenin and subsequent cell growth [111].

The transgenic mice expressing hSiglec-9-hIg2 transplanted MM46-MUC1 mammary tumor cells intraperitoneally suppressed proliferation of MM46-MUC1 tumor cells, as compare with non-transgenic mice [88]. The anti-tumor potential was not observed in the transgenic mice bearing MM46 cells without forcibly expressing MUC1. Histopathological and blood biochemical abnormalities were not observed in the transgenic mice. Furthermore, there was no significant effect on a number of lymphocyte homing into lymph nodes at least. Altogether, these findings suggested that hSiglec-9-hIg2 may be a medical agent against the tumor expressing MUC1 without side effects on the immune system [88].

Purified hSiglec-9-hIg2 was not cytotoxic for MM46-MUC1 cells in vitro [88]. However, the proliferation of MM46-MUC1 cell in the transgenic mice was lower than that in non-transgenic mice. Suppression of the proliferation of MM46-MUC1 cell in vivo may be due to inhibition of the MUC1 downstream signal transduction through direct suppression of MUC1 expression and/or inhibition of MUC1 binding to CD33-related Siglec by hSiglec-9-hIg2. On the other hand, it was reported that binding of the soluble form of Siglec-9 to the MUC1 induced recruitment of β-catenin and subsequent cell growth in vitro rather than suppression of the tumor growth, which results disagreed with our ones in vivo [111]. The discrepancy might be explained as follows. The effects in cell proliferation by hSiglec-9-hIg2 may depend on various factors in vivo such as the presence of endogenous CD33-related Siglec or similar molecules, and their localization and concentration on immune cells.

Histological examination revealed that the viable CD3-positive T lymphocytes were infiltrated through the tumor ascites in the transgenic mice, whereas aggregations of degenerative CD3-positive cells were often observed in the non-transgenic mice [88]. In addition, the proliferative activities of transgenic splenic T lymphocytes were slightly higher than non-transgenic T lymphocytes. It was reported that bindings of Siglec-9 to prohibitins negatively regulated TCR signaling [112]. These findings indicated that hSiglec-9-hIg2 inhibits the binding of CD33-related Siglec similar to human Siglec-9 and prohibitins, leading to the histopathological difference in the T cell infiltration in the ascites. In addition, it was reported that cancer-associated MUC1 disturbed proliferation of T lymphocytes and caused apoptosis of T lymphocytes [113,114]. It seemed that suppression of MUC1 expression indirectly affected activity and proliferation T cells in the tumor. Taken together, hSiglec-9-hIg2 could suppress proliferation of MM46-MUC1 tumor cells and might be a candidate as a new therapeutic agent targeting MUC1.

## 5. Conclusions and Future Directions

Several soluble forms of cell adhesion molecules are able to provide anti-viral, bacterial and tumor benefits by their direct binding to the counterpart molecules and/or through indirect effects of the immune mechanism in transgenic animals. Although some of the molecules cause severe side effects in the transgenic animals, it seems that there may be almost no risk for their therapeutic usage. For preventive medicine of infectious diseases, the soluble forms of nectin-1, HVEM and Siglec-9 that directly bind to pathogens may be useful agents, but so far there is no apparent evidence of their potential for treatment of infectious disease. However, suppression of manifestation of the disease was observed in transgenic mice expressing a soluble form of HVEM or Siglec-9 by which may be stimulated the immune response. In addition, anti-GBS benefit may be enhanced through the innate immune mechanism utilizing the Fc portion of the soluble form of human Siglec-9.

For elucidation of the mechanism for these possible effects and therapeutic possibilities of soluble forms of CAMs, further investigation is needed in animal models. Furthermore, genome editing may provide a point mutation in the cell surface protein genes to produce their soluble form of proteins for development of disease-resistant animals.
